# Special Issue: “Evolution, Ecology and Diversity of Plant Virus”

**DOI:** 10.3390/v15020487

**Published:** 2023-02-09

**Authors:** Mengxue Yin, Wenxing Xu

**Affiliations:** 1 National Key Laboratory for Germplasm Innovation & Utilization of Horticultural Crops, Wuhan 430070, China; 2 College of Plant Science and Technology, Huazhong Agricultural University, Wuhan 430070, China

The next-generation sequencing method was developed in the second half of the 2000s and marked the beginning of high-throughput sequencing (HTS) analyses of viral communities [1,2]. With this approach, numerous novel virus communities have been discovered and characterized in recent years, which expanded our understanding of the evolution, ecology, and diversity of plant viruses [3,4,5]. This Special Issue contains four original studies about the characterization of novel viruses revealed by HTS, evolution, ecology, and the biodiversity of plant viruses from 2021 to 2022.

With the HTS approach, a novel badnavirus, provisionally designated chinaberry tree badnavirus 1 (ChTBV1), was identified from chinaberry that has been cultivated in China for use in traditional medicines [6]. Thereafter, a sensitive and robust method relying on a loop-mediated isothermal amplification (LAMP) assay was developed to detect ChTBV1 from Chinaberry tree leaves [6].

Genetic recombination between viral isolates has been known to be one of the sources of speciation [7] and is a major source of viral variability, which benefits viral communities in the expansion of their host ranges, the alteration of transmission vector specificities, and increases in virulence and pathogenesis [8,9]. Here, one original study revealed a new speciation of an emergent ssDNA plant virus, tentatively named ‘Chenopodium leaf distortion virus’ (CLDV) associated with *Chenopodium album* via recombination; the study suggests that weeds serve as mixing vessels for begomoviruses, thereby facilitating recombination [10].

Moreover, the spatiotemporal spread of a plant virus, cherry leaf roll virus (CLRV), was studied using a Bayesian phylodynamics framework based on the coat protein gene sequences of 81 viral isolates collected from 5 different countries. It revealed that Germany played an important role in CLRV transmission, which is consistent with the trade of cherry [11].

The genome sequence of several isolates of apple rubbery wood virus 2 (ARWV-2) and citrus virus A (CiVA), two negative-sense single-stranded RNA viruses, infecting pear trees grown in China was characterized by using the HTS approach combined with conventional RT-PCR assays. The results show that genome-wide nt sequence identities were above 93.6% among the ARWV-2 isolates and above 93% among CiVA isolates, and sequence diversity occurred in the 5′ untranslated region of the ARWV-2 genome and the intergenic region of the CiVA genome [12].

This Special Issue informs us of up-to-date knowledge that genetic recombination, spatiotemporal spread, and host plants have largely shaped viral communities, including their speciation, evolution, ecology, and diversity of plant viruses. Altogether, this will help us better understand plant viruses and the development of a more efficient strategy for controlling plant viral diseases.
